# Outcomes of Extravesical Versus Intravesical Ureteral Reimplantation

**DOI:** 10.1100/tsw.2004.68

**Published:** 2004-08-26

**Authors:** Leah P. McMann, Byron D. Joyner

**Affiliations:** Department of Surgery, Urology Service, Madigan Army Medical Center, Tacoma, WA 98431, USA

## Abstract

Purpose: The purpose of our study was to examine outcomes and compare length of stay after extravesical and intravesical ureteral reimplantation at our institution. Materials and Methods: Retrospective review was performed of 30 patients (55 ureters) with vesicoureteral reflux who underwent either the Cohen (intravesical) cross-trigonal procedure or the extravesical (detrusorrhaphy) approach. Each patient had documented follow-up consisting of a postoperative renal ultrasound and/or a voiding cystourethrogram (VCUG). Inclusion criteria was the presence of primary vesicoureteral reflux. Exclusion criteria were patients who had undergone a previous repair and patients in whom results of neither the renal ultrasound nor the VCUG were available. Results: There were no significant cases of obstruction or wound infection with either approach. Two patients who underwent the extravesical approach had persistent reflux on VCUG three months postoperatively, but both resolved by fifteen months. Average length of stay was only 3.00 ± 1.33 days for the extravesical approach, compared to 5.36 ± 1.75 days for the intravesical approach ( P = .0003 ). Conclusions: Given that by fifteen months success rates were the same with either approach, the extravesical approach is comparable to the intravesical technique and is a viable option in terms of outcome and economics given the shorter length of hospital stay.

## INTRODUCTION

In the United States, intravesical ureteral reimplantation such as the Leadbetter-Politano or the Cohen cross-trigonal procedure, is currently the most popular way to manage vesicoureteral reflux. The extravesical technique was first described in Europe by Lich in 1961 (1) and subsequently in the United States by Gregoir in 1964. (2) With this technique, the bladder is left intact and it is generally associated with the advantages of minimal hematuria, infrequent urinary leaks, reduced bladder spasms, and potentially shorter hospital stays. (3) We perform both the Cohen cross-trigonal (intravesical) and the detrusorrhaphy (extravesical) procedures at our institution, and have compared the two approaches with respect to postoperative results, complications, and length of hospital stay.

## MATERIALS AND METHODS

Cases of ureteral reimplantation performed at our institution between 1996 and 2000 were retrospectively reviewed. Inclusion criteria were the presence of primary vesicoureteral reflux. Exclusion criteria were patients who had undergone a previous repair and patients in whom the results of neither a postoperative renal ultrasound nor a postoperative voiding cystourethrogram (VCUG) were available. The intravesical procedure consisted of a cross-trigonal tunnel sewn in place by interrupted sutures, as previously described by Cohen. The extravesical procedure consisted of mobilization of the ureter extravesically to the ureterovesical junction, incision of the detrusor around the ureter, ureteral advancement into the submucosal tunnel, and reapproximation of the detrusor over the ureter. A total of 38 patients were initially identified as having undergone ureteral reimplantation during that time period. Two of the 38 were revisions, and six of the 38 were followed up at outside institutions so neither the results of the renal ultrasound nor the VCUG were obtained. Therefore, 30 patients (55 ureters) underwent repair of primary reflux and completed their follow-up evaluation at our institution. Reflux was graded according to the International Reflux Study Group grading system. Patient age ranged from eleven months to 23 years (mean age, 8.56 years). Two patients had unilateral duplication and two patients had bilateral duplicated systems. Twelve patients (22 ureters) underwent the Cohen cross-trigonal (intravesical) approach. Eighteen patients (33 ureters) underwent the detrusorrhaphy (extravesical) approach. All 30 patients had documentation of their postoperative evaluation with either a renal ultrasound at six weeks postoperatively and/or a VCUG at three months postoperatively. Length of stay data was subjected to a statistical evaluation using the Student's t test. .

## RESULTS

No significant cases of obstruction or wound infection were identified with either approach. The results of renal ultrasound for evaluation of the upper tracts showed three of the 30 with mild hydronephrosis, two who had undergone the intravesical approach, one who had undergone the extravesical approach. On postoperative VCUG, two patients had persistent low grade reflux. Both cases of persistent reflux resolved on repeat VCUG at 15 and 10 months, respectively. The average length of hospital stay for patients who underwent extravesical reimplantation was 3.00 ± 1.33 days compared to 5.36 ± 1.75 days for the patients who underwent the intravesical procedure ( $P = .0003$ ). There was no significant morbidity associated with either approach.

## DISCUSSION

Although the intravesical technique for the surgical management of vesicoureteral reflux is more popular in the United States, the extravesical technique offers the advantage of an overall shorter hospital stay likely due to less morbidity associated with leaving the bladder intact, less hematuria, and fewer bladder spasms. (3) The extravesical approach first described by Lich and then Gregoir showed resolution of reflux in 329 (96%) of 362 cases and persistence of reflux in 12 cases (14%). Their average hospital stay was five days. In 1971, Daines and Hodgson described a modified extravesical approach to include advancement of the ureteral orifice to provide a longer submucosal tunnel, noting a success rate of 98% and a hospital stay of two to four days. (4) However, it has not gained widespread popularity in the US due to the reporting of a high failure rate (58%) by Hendren in 1974 (5) and concerns about postoperative voiding inefficiency. In 1987, Zaontz et al described their series of 120 renal units treated using the extravesical approach and cited resolution of reflux in 111 ( 93% ) and persistence in 9 ( 7 %). (6) In

1995, Ellsworth and Merguerian compared the Leadbetter-Politano (intravesical) and the detrusorrhaphy (extravesical) procedure with respect to outcome, postoperative pain control, and length of stay and noted a success rate of 94.7% for the extravesical approach and 95.3% for the intravesical approach. (7) Also in 1995, Fung et al examined voiding efficiency after intravesical and extravesical unilateral ureteral reimplantation and found no difference in postoperative voiding efficiency between the two approaches. In their series, patients who underwent the extravesical bilateral technique had a statistically significantly higher proportion of patients with transient voiding inefficiency, but fully recovered with time and there was no significant difference in the rate of resolution of reflux between the intravesical and extravesical groups. (8) The authors concluded that the combination of reduced morbidity, potentially excellent outcome, and relative ease in gaining technical expertise makes the extravesical unilateral procedure an excellent alternative to the traditional intravesical procedure. (8) The most common intravesical technique performed at our institution between 1996 and 2000 was the Cohen cross-trigonal procedure. Our study compares the Cohen intravesical ureteroneocystostomy and the extravesical detrusorrhaphy with respect to outcomes and length of hospital stay. Our initial success rate of 89% using the extravesical approach was slightly lower than that reported by Lich, (1) Gregoir, (2) Zaontz, (6) and Ellsworth. (7) However, both cases of persistent reflux were transient, and resolved by fifteen months postoperatively. Patients undergoing the extravesical approach had a hospital stay which was on average two days shorter than those undergoing the intravesical approach. At our institution, this translates to avoiding an additional cost of $4861.32 per patient, since our cost per day for a urology inpatient is currently $2430.66.

## CONCLUSIONS

Based on our experience at this institution, extravesical reimplantation is a reliable technique and is comparable to intravesical reimplantation with regard to long-term outcome. Moreover, the extravesical approach provides an easier postoperative course with little morbidity for the patient. Because the bladder is left intact, gross hematuria rarely occurs and the patient has fewer bladder spasms. Less morbidity is associated with a shorter hospital stay, leading to a substantial reduction in cost per day for a urology inpatient at our institution.
